# Virus Identification for Monkeypox in Human Seminal Fluid Samples: A Systematic Review

**DOI:** 10.3390/tropicalmed8030173

**Published:** 2023-03-14

**Authors:** Joshuan J. Barboza, Darwin A. León-Figueroa, Hortencia M. Saldaña-Cumpa, Mario J. Valladares-Garrido, Emilly Moreno-Ramos, Ranjit Sah, D. Katterine Bonilla-Aldana, Alfonso J. Rodriguez-Morales

**Affiliations:** 1 Escuela de Medicina, Universidad Cesar Vallejo, Trujillo 13007, Peru; 2 Facultad de Medicina Humana, Universidad de San Martín de Porres, Chiclayo 15011, Peru; 3 Centro de Investigación en Atención Primaria en Salud, Universidad Peruana Cayetano Heredia, Lima 15102, Peru; 4 South American Center for Education and Research in Public Health, Universidad Norbert Wiener, Lima 15108, Peru; 5 Oficina de Epidemiología, Hospital Regional Lambayeque, Chiclayo 14012, Peru; 6 División de Revisiones Sistemáticas y Meta-Análisis, Tau-Relaped Group, Trujillo 13007, Peru; 7 Tribhuvan University Teaching Hospital, Institute of Medicine, Kathmandu 44600, Nepal; 8 Department of Public Health Dentistry, Dr. D.Y. Patil Dental College and Hospital, Dr. D.Y. Patil Vidyapeeth, Pune 411018, Maharashtra, India; 9 Research Unit, Universidad Continental, Huancayo 12000, Peru; 10 Grupo de Investigación Biomedicina, Faculty of Medicine, Fundación Universitaria Autónoma de las Américas-Institución Universitaria Visión de las Américas, Pereira 660003, Colombia; 11 Master of Clinical Epidemiology and Biostatistics, Universidad Cientifica del Sur, Lima 15067, Peru; 12 Gilbert and Rose-Marie Chagoury School of Medicine, Lebanese American University, Beirut 1102, Lebanon

**Keywords:** monkeypox, seminal fluid, samples, orthopoxvirus, monkeypox virus

## Abstract

Public health officials around the world are extremely concerned about the global outbreak of monkeypox (MPX), which has been claimed to have originated in Africa. As a result, studies into the origins and reasons behind the outbreak’s rapid spread have been sped up. The goal of the current investigation is to determine whether the monkeypox virus (MPXV) is present in seminal fluid samples from MPX cases that have been verified. Up until 6 January 2023, PubMed, Scopus, Web of Science, Embase, and ScienceDirect databases were used to conduct a thorough evaluation of the literature. The search technique returned a total of 308 items. Fourteen studies reporting the presence of MPXV in the seminal fluid of MPX-confirmed cases were included after the duplicates (*n* = 158) and searches by title, abstract, and full text were eliminated. In 84 out of the 643 confirmed MPX cases (13.06% or *n* = 643), MPXV was discovered in seminal fluid. Reverse transcriptase polymerase chain reaction (RT-PCR) was used to identify MPXV, and samples taken from skin lesions (96.27%), pharynx or oropharynx (30.48%), and blood all had higher positivity rates than other samples (12.44%). Additionally, 99.85% of respondents were male with a mean age of 36, 98.45% engaged in MSM (men who have sex with men) sexual conduct, and human immunodeficiency virus (HIV) accounted for 56.9% of all STD cases. This study offers proof that MPXV can be found in the seminal fluid of MPX sufferers. Our data imply that MPXV transmission is a possibility in these samples and that MSM are more vulnerable to it. The creation of hygienic standards is essential for the early identification of MPX cases.

## 1. Introduction

The 1979 eradication of smallpox certified by the World Health Organization (WHO) resulted in the cessation of routine smallpox vaccination in most countries. It is estimated that more than 70% of the world’s population is no longer protected against smallpox or by cross-immunity against closely related orthopoxviruses such as monkeypox [1].

During the COVID-19 pandemic, the fast spread of monkeypox (MPX) cases in many nations throughout the world has aroused interest and worry about the illness globally [2]. On 23 July 2022, the Director General of the WHO designated the MPX outbreak as a Public Health Emergency of International Concern [3].

The monkeypox virus (MPXV) is a zoonotic virus that causes MPX [4], A double-stranded DNA virus known as MPXV belongs to the family Poxviridae’s genus Orthopoxvirus and is only found in a small number of endemic countries in Central and West Africa [5]. In 1958, this virus was discovered in captive monkeys, and in 1970, it was discovered in a child in the Democratic Republic of the Congo [6]. In addition, the virus has two variants: the Congo Basin clade and the West African clade.

MPX has an initial prodromal phase with variable symptoms such as fever, lymphadenopathy, fatigue, and malaise [7]. Most patients present with focal, more abundant, or more severe lesions in the genital, anorectal, and oropharyngeal regions, possibly reflecting high levels of virus exposure during sexual intercourse [8]. 

Most of the MPX cases detected during the 2022 outbreak were men who have sex with men (MSM) who engaged in high-risk sexual behavior [9]. The MPXV transmission methods are developing quickly. Close sexual contact with one or more MPX lesions on the skin or mucosal surfaces (such as the oropharynx or anorectum) of a person with MPX has been shown to be the predominant mechanism of transmission in the present outbreak [10].

The temporal correlation between symptoms and sexual contact, as well as the co-occurrence of main lesion sites with those of sexual contact, are evidence that MPXV is transmitted through semen during sexual activity [11,12]. According to a recent study, the viral DNA quantities in semen were lower than those found in skin lesions [13].

Diagnostic methods are crucial for infectious disease surveillance and management. There are currently accessible MPXV serological, sequencing, and nucleic acid amplification assays [14]. Polymerase chain reaction (PCR) and sequencing are frequent MPXV diagnostic techniques. Skin lesion swabs, oropharyngeal swabs, anal swabs, urethral swabs, conjunctival swabs, and semen have all revealed the presence of MPXV [15].

Currently, the spread of MPXV continues to evolve, and as new scientific evidence becomes available, a better approach to this disease will be guided. Studies report that most cases of MPX are transmitted through sexual contact, so there is concern about the possible transmission of the virus between seminal fluids [16]. The present study aims to identify MPXV in seminal fluid samples from MPX cases.

## 2. Materials and Methods

### 2.1. Protocol Registration

This systematic review was registered on PROSPERO (International Prospective Register of Systematic Reviews) with registration number CRD42022372578.

### 2.2. Eligibility Criteria

We included peer-reviewed articles published in research formats such as case reports, case series, and observational studies (nonrandomized cohort and intervention studies) to investigate the identification of the monkeypox virus in seminal fluid samples from confirmed cases. The articles, which comprised everything published up to 6 January 2023, had no language restrictions. Study designs such as editorials, letters to the editor, randomized clinical trials, narrative reviews, systematic reviews, and conference proceedings were excluded.

### 2.3. Sources of Information and Search Techniques

We carefully searched the ScienceDirect, PubMed, Scopus, Web of Science, and Embase databases. Such as “Monkeypox” and “Semen”, we used restricted terms and their Thesaurus mesh and Emtree (Table S1, GitHub repository). On 6 January 2023, the searches were finished, and two separate researchers independently assessed the results.

### 2.4. Study Selection

Two investigators (J.J.B. and D.A.L.F.) used management software to create a database from their electronic searches, and it was duplicate-free. The two researchers finished the Rayyan QCRI screening method after that (H.M.S.C. and D.K.B.A.). They evaluated the titles and abstracts that the search returned individually, chose those that appeared to meet the criteria for inclusion, and, if necessary, studied the entire text. Before bringing in a third investigator to mediate any disagreements, the investigators first discussed their differences and reached a resolution.

Before deciding whether to include the reports, the authors (R.S., M.J.V.G., A.J.R.M., and E.M.R.) carefully examined the full-text reports and inclusion criteria.

### 2.5. Outcomes

The main result was to test for monkeypox virus presence in seminal fluid samples from confirmed cases of monkeypox.

### 2.6. Data Collection Process and Data Items

Three researchers (D.A.L.F., R.S., and J.J.B.) independently extracted the data from the selected papers and entered it into an Excel spreadsheet. From the selected studies, details about the author, publication date, study design, country, number of monkeypox cases, number of monkeypox-positive semen samples, age, sex, risk factors, recent sexual exposure, STIs, other monkeypox-positive samples, monkeypox diagnosis, clinical manifestations, site of skin lesions, and outcome were taken.

A fourth researcher handled any disagreements concerning the inclusion of studies and verified that the list of publications and data extractions were free of duplicate articles or other content.

### 2.7. Tables

The tables of results presented in this study are in a cell format hosted on the GitHub repository (https://github.com/jbarbozameca/Tropicalmed-2098479; accessed on 14 February 2023). They can be accessed directly through the link.

## 3. Results

### 3.1. Study Selection

**Figure 1:**
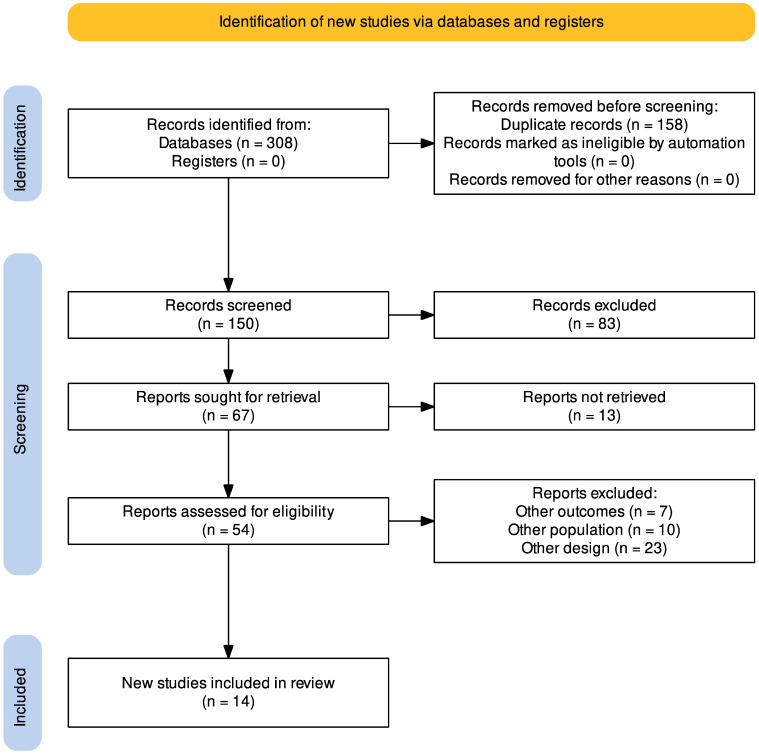
Process for choosing studies, according to the PRISMA flowchart.

There were 308 articles altogether in the results of the search method. The selection process is illustrated in the PRISMA flow chart (Preferred Reporting Items for Systematic Reviews and Meta-Analyses). One hundred and fifty articles were evaluated by the authors after duplicates (*n* = 158) were eliminated from the list. Fifty-four publications were chosen for full-text reading after the titles and abstracts had been filtered, and fourteen of those were deemed suitable for this systematic review (Figure 1) [11,17,18,19,20,21,22,23,24,25,26,27,28,29].

### 3.2. Study Characteristics

Table S2 (GitHub repository) provides a summary of the main characteristics of the included studies [11,17,18,19,20,21,22,23,24,25,26,27,28,29]. Fourteen studies were part of the systematic review and were published between 1 January and 31 December 2022 [11,17,18,19,20,21,22,23,24,25,26,27,28,29]. There were 643 confirmed cases of MPX reported, and 13.06% of them (*n* = 84) were found in seminal fluid [11,17,18,19,20,21,22,23,24,25,26,27,28,29].

### 3.3. Demographic Characteristics and MPXV-Positive Samples

Of the cases registered with MPX (*n* = 643), 99.85% were men (*n* = 642) with an average age of 36 years, and 98.45% had a sexual behavior of being men who have sex with men (*n* = 633) [11,17,18,19,20,21,22,23,24,25,26,27,28,29]. The most prevalent sexually transmitted infections (STIs) were 56.9% HIV (*n* = 366) [11,17,20,21,22,23,24,25,26,28], 5.91% syphilis (*n* = 38) [21,22,24], and 5.29% gonorrhea [18,21,24]. All reported cases with MPX had a risk of MPXV transmission by sexual contact. In addition, MPXV was detected by reverse transcriptase polymerase chain reaction (RT-PCR) [11,17,18,19,20,21,22,23,24,25,26,27,28,29]. The most frequent locations of positive samples used for MPXV diagnosis were: 96.27% skin lesions (*n* = 619) [11,17,18,19,20,21,22,23,24,25,26,27,28,29], 30.48% pharynx or oropharynx (*n* = 196) [17,18,19,20,21,22,23,24,26,28,29], 13.06% semen [11,17,18,19,20,21,22,23,24,25,26,27,28,29], and 12.44% blood (*n* = 80) [11,17,18,19,21,22,23,25,27,28] (Table S2, GitHub repository).

### 3.4. Clinical Manifestations, Localization of Skin Lesions, and Outcome

The most frequent clinical manifestations in patients confirmed with MPX were 58.01% fever (*n* = 373) [11,17,18,19,20,21,22,23,24,26,27,28,29], 51.79% lymphadenopathy (*n* = 333) [11,17,18,19,20,21,23,26,27,28,29], and 26.75% headache (*n* = 172) [17,20,21,23,24,28] (Table S3, GitHub repository). The most frequent locations of the lesions were 68.12% perianal region (*n* = 438) [11,17,18,21,22,24,25,26,28,29], 67.65% genitalia (*n* = 435) [11,18,19,20,21,22,24,25,27,28], 46.50% trunk (*n* = 299) [21,23,24,26,27], 24.88% face (*n* = 160) [21,26,28], and 10.89% upper and lower extremities (*n* = 70) [11,20,21,22,24,26,28,29]. The evolution of these lesions was asynchronous. Without specific treatment, all patients recovered completely [11,17,18,19,20,21,22,23,24,25,26,27,28,29] (Table S3, GitHub repository).

## 4. Discussion

### 4.1. Main Findings and Current Epidemiological Data

In this systematic review, MPXV was detected in seminal fluid samples from 13.06% of confirmed cases. Additionally, 99.85% were men with an average age of 36 years, 98.45% had a sexual behavior of being men who have sex with men and the most prevalent sexually transmitted infection was HIV in 56.9%.

The global MPX outbreak has been classified as a public health emergency of international concern by the WHO [30]. 85,565 confirmed cases of MPX have been reported in 110 countries as of 5 February 2023 [31]. 96.6% (71,946/74,479) of patients with data are male, and among cases with reported sexual orientation, 84.4% (25,946/30,733) were identified as gay, bisexual, or other males who have sex with men (MSM), according to WHO epidemiological data [32].

The epidemiology of MPX is complex and varied; this disease is endemic in Africa but has now emerged in different countries around the world. The transmission dynamics of the virus are changing, and according to recent scientific reports, the population with a risk behavior of being men who have sex with men is being affected.

### 4.2. Detection of MPX in Seminal Fluid Samples from Confirmed Cases

The MPX virus was found in seminal fluid samples from 13.06% of confirmed cases. According to the study by Reda A et al. [16], MPXV can be identified in semen samples from the first day and up to 19 days after symptom onset. The replication capacity of MPXV particles found in seminal samples was also tested. The culture of MPXV was successful. MPXV is highly prevalent in seminal samples from MPX cases, supporting the importance of the sexual transmission of the disease. However, further research is needed to shed more light on the replication potential of these particles.

According to Thornhill JP et al. [21], deoxyribonucleic acid (DNA) of MPXV was detectable by PCR in the seminal fluid in 29 out of 32 cases. However, it is unknown whether the viral DNA discovered in these samples was capable of replication, so it must be determined whether semen can spread the infection. Reports of clusters associated with sex parties or saunas highlight the potential importance of sexual interactions as a promoter of transmission. International travel and attendance at large gatherings, along with on-site sexual activities, may explain the global spread of amplified MPX infections through sexual networks.

Similar results were reported by Raccagni AR et al. [25]. In 22 of the 36 individuals, MPXV was detected in the seminal fluid. In addition, all reported high-risk sex in the three months prior to diagnosis was considered, including unprotected sex with more than 10 partners.

According to the Centers for Disease Control and Prevention (CDC) [33], MPXV DNA has been found by PCR in the semen of infected men, and semen has generated a replication-competent virus, but no case has been documented in which semen exposure was the only conceivable method of MPXV transmission. Transmission by receptive anal, vaginal, or oral sexual contact is particularly difficult to measure, as such exposure usually occurs in the context of sexual intercourse involving skin-to-skin contact. Viral DNA concentrations in semen were found to be lower than those in skin lesions. Thus, semen exposure could plausibly transmit the disease, but the data are insufficient to definitively confirm this exposure as a source of infection.

In animal models, testes are target organs of many viral infections, such as Zika virus [34], Ebola virus [35], Marburg virus [36], and Crimean-Congo hemorrhagic fever virus [37]. Liu J et al. [38], in their study on MPXV infection and its persistence in the testes of crab macaque monkeys, found MPXV antigen and messenger RNA in seminiferous tubules and the lumen of epididymal ducts. In addition, MPXV can be disseminated in semen during the acute and convalescent stages of the disease in crab macaques.

Recent reports have shown the detection of viruses in the semen of men infected with hitherto unknown sexually transmitted viruses (chlamydia, gonorrhea, syphilis, acquired immune deficiency syndrome, and genital herpes), which has attracted the attention of infectious disease specialists, public health officials, and the media [39]. Most STI-causing microorganisms are associated with male reproductive tract problems, such as genital lesions, semen infections, prostatitis, urethritis, epididymitis, and orchitis [40].

### 4.3. Clinical Characteristics of Patients with MPX

Monkeypox virus was detected by PCR. Most of the positive samples used for MPXV diagnosis were extracted from skin lesions (96.27%), pharynx or oropharynx (30.48%), semen (13.06%), and blood (12.44%).

According to a systematic review investigation conducted by León-Figueroa DA et al. [13], the samples taken for diagnosis that tested positive for MPXV were 91.85% from skin lesions, 20.81% from the oropharynx, 3.19% from blood, and 2.43% from seminal fluid. In another study, skin specimens had the highest PCR positive rate for MPXV (89%), followed by anogenital/rectal specimens (74.3%). In contrast, the positivity rates in nasopharyngeal (62.4%), urine (21.1%), and blood/plasma (14.3%) samples were lower [16].

HIV (56.9%), syphilis (5.91%), and gonorrhea (5.29%) were the most prevalent sexually transmitted infections. In their case series, Thornhill JP et al. [21] found that 218/528 of the MPX individuals (21.8%) had HIV. This is comparable to what Raccagni AR et al. [25] reported in Italy, where 15.36% of MPX cases carried HIV. MPX is currently spreading outside endemic African countries, and most of those affected are homosexual and bisexual men in linked sexual networks [41]. A systematic review study conducted at the beginning of the 2022 outbreak reported 4222 patients with MPX, of which 22.48% were HIV-positive [12].

Ortiz-Saavedra B et al. reported in their study that 40.32% of cases with MPX had HIV coinfections [42]. Coinfections with these two viruses are very harmful, as it can aggravate the symptoms of both diseases and make them more difficult to treat. Since their immune systems are compromised, people with HIV are more susceptible to some diseases, including MPX. It is therefore important to assess the relationship between MPX and sexually transmitted diseases, which is generating growing concern, because it is believed that MPXV could be transmitted through sexual contact.

The clinical signs of MPX are similar to those of smallpox, with nonspecific clinical features such as fever, chills, myalgia, headache, lethargy, and lymphadenopathy, followed by a vesicular–pustular rash with an incubation period ranging from 5 to 21 days [4,18].

In individuals whose MPX results were verified, fever (58.01%), lymphadenopathy (51.79%), and headache (26.75%) were the most frequently occurring clinical symptoms. This is comparable to what Benites-Zapata VA et al. [43] observed in 1958 individuals, who had the most common symptoms be rash (93%), fever (72%), pruritus (65%), and lymphadenopathy (62%). A common MPX presentation includes a fever, lymphadenopathy, headache, and other bodily aches and symptoms [44]. It is important to note that symptoms can vary in severity, and some patients may not have obvious symptoms. It is important to seek medical attention if MPX or any other infectious disease is suspected.

We discovered that the perianal region (68.12%), genitalia (67.65%), trunk (46.50%), face (24.88%), and upper and lower extremities were the most frequently affected areas (10.89%). This is comparable to the findings of Cassir N et al. [45], who discovered that, out of 136 patients, the genitalia (68 patients, 53%), perianal region (65 patients, 49%), and oral/perioral area were the most common lesion locations (22 patients, 17%).

A systematic review study reported that the most frequent lesion locations were perianal (28.64%), genital (32.52%), oral (19.23%), trunk (15.16%), and upper and lower extremities (11.63%) [12]. Similar results were reported in a case series study in which, of 528 cases of MPX, 95% had a skin rash, 73% had anogenital lesions, and 41% had mucosal lesions [21].

All patients had a full recovery without any specific treatment, because the progression of these lesions was asynchronous. Some of the patients were being treated for sexually transmitted diseases and others for pain or stinging from the lesions.

Although there is currently no viable treatment for MPX, the outbreak’s growth has raised public concerns, particularly given that COVID-19’s outbreak is still running strong [12]. As a result, tecovirimat, cidofovir, and brincidofovir are the medications approved and utilized for the antiviral treatment of MPX [46].

The antiviral tecovirimat prevents the release, spread, and pathogenicity of enveloped viruses by inhibiting the activity of the protein p37 [47]. MPXV DNA synthesis is slowed by cidofovir [48]. Brincidofovir is effective against viruses with double-stranded DNA and works as an antiviral by entering infected cells [49]. Immunity against MPXV is equal to that against smallpox [50].

The global situation urgently requires evidence of the efficacy and safety of antivirals against MPX [51]. Three potential bioavailable drugs, tecovirimat, brincidofovir, and cidofovir, have been tested in the United States of America (USA) and Europe to treat MPXV [52]. However, no clinical trials have been conducted to evaluate their efficacy and safety in patients with MPX.

The safety of tecovirimat, brincidofovir, and cidofovir has been previously established in healthy human subjects [53]. Animal trials have demonstrated their efficacy. Based on research, tecovirimat would be the ideal choice for MPX administration because of its simplicity of administration (oral drug), lower side effects, and past treatment success [51].

Although there is currently insufficient data on antiviral therapies for MPX, clinical management guidelines are crucial tools to guide clinical decision-making and standardize the best care [54]. Guidelines should be easily accessible, of high quality, and inclusive of vulnerable patient populations. Patients will benefit from the standardization of treatment, which can aid in conducting the necessary multicenter studies for drugs and vaccines. The increase in MPX cases in recent decades underscores the importance of providing clinicians worldwide with clinical management guidelines to guide therapy and improve patient care and outcomes.

Vaccination is important to contain the outbreak of MPX. The U.S. Food and Drug Administration has licensed two vaccinations that can prevent MPXV: ACAM2000 (a live attenuated, replicating vaccine) and JYNNEOS (a live attenuated, non-replicating vaccine) [55].

Due to the safety provided by these vaccines, JYNNEOS has been selected for the current MPX outbreak [56]. It is licensed as a two-dose series to prevent MPX in persons 18 years of age and older. For complete immunization, two doses of the vaccine should be administered at least 28 days apart. Two weeks after the second dose of vaccination, the highest level of protection against MPX is guaranteed. ACAM2000 carries a high risk of significant adverse effects and should not be administered to patients with compromised immune systems, skin problems, heart disease, or who are pregnant or suspecting pregnancy [57]. The CDC recommends JYNNEOS as the primary vaccine, because it has fewer potential adverse effects than ACAM2000.

MSM are clearly the most affected by the current MPX outbreak. Consequently, public health authorities have collaborated to immunize high-risk individuals against MPX. Due to chronic underfunding, which has been exacerbated by the COVID-19 pandemic, sexual health clinics have served as a logical, low-barrier setting for individuals seeking MPX prevention. However, they may not be prepared for the challenge of responding to an emerging infectious disease while providing their usual services [58].

### 4.4. Future Perspectives on the MPX

As the COVID-19 pandemic continues to rage worldwide, MPX cases are increasing in nonendemic countries, raising global public health concerns about the possibility of a new pandemic. Mutations in coding sections of the viral genome may have resulted in fitness adaptation, enhanced immune evasion mechanisms, and increased transmissibility of MPXV [59]. Several causes, such as the decline in cross-protective herd immunity (the discontinuation of smallpox vaccination), deforestation, civil strife, refugee relocation, agriculture, increasing global interconnectedness, and even climate change, may accelerate the emergence of this disease. With this in mind, healthcare professionals must update their knowledge of the diagnosis, prevention, and clinical management of PXM [60].

Therefore, it is important to improve the integrated surveillance of MPXV, strengthen the diagnostic methods, train health personnel, employ public education programs, provide funding for research on these emerging diseases, and guide a “One Health” approach [61].

In recent years, outbreaks of MPX have occurred in several countries in Africa, as well as in occasional cases in other regions of the world. As with many infectious diseases, the future of MPX will depend on several factors, including the continued emergence of new strains, the success of public health measures to control outbreaks, and the availability of effective vaccines and treatments.

One possible future evolution of MPX is the development of new treatments and vaccines. Currently, there is no specific treatment for monkeypox, and the smallpox vaccine is believed to provide some level of protection against the disease. However, the development of new treatments and vaccines, particularly those that are more effective against new strains of the virus, could have a significant impact on the control of future outbreaks.

Another important factor will be the continued surveillance and monitoring of human and animal cases of monkeypox to better understand disease transmission patterns and to identify any potential new outbreaks early. This will require continued investment in public health infrastructure and surveillance systems, as well as collaboration between countries and international health organizations.

### 4.5. Limitations and Strengths

More research is required on the identification of MPXV in seminal fluid, which will provide sufficient proof that this disease is spread through sex, as information on the transmission of MPXV is continually changing. The current investigation provides proof that MPXV was found in the seminal fluid of patients with verified MPX.

Although the present study’s methodology included case report and case series investigations, which could lead to information heterogeneity, it meticulously adhered to the PRISMA standards.

In addition, each included study was evaluated by two or more authors independently. The evidence reported serves as a basis for future research as more evidence on the identification of MPXV in seminal fluid is reported.

### 4.6. Relevance of Findings in Public Health

Understanding the transmission, replication, and transfer of MPXV through the seminal fluid is essential to comprehending the epidemiology of the present outbreak that has spread to numerous nations. The majority of infections are acquired by men who have sex with other men, despite an ongoing study into the exact causes and means of MPX transmission [62].

As a result, the evidence presented in the current study focuses on the transmission of MPXV via sexual interactions. Therefore, efforts should be made globally to enhance intervention strategies to reduce the sexual transmission of MPXV among various demographic groups [63].

From a public health standpoint, the findings related to MPX are very important, as knowledge of the disease and its transmission can help prevent outbreaks and protect vulnerable populations. Some key findings related to MPX that have public health relevance include transmission, clinical presentation, treatment and vaccine development, and global distribution.

### 4.7. Prevention Measures

The pillars for minimizing the spread of the disease include rapid identification and effective isolation of patients, use of personal protective equipment by healthcare personnel, hand cleaning, and thorough contact tracing, including the follow-up of secondary cases throughout the incubation period [64].

Public health authorities play an essential role in rapidly identifying and isolating infected individuals, treating them, and administering the necessary vaccines. Innovative approaches to “close contact” surveillance are highly desirable to prevent further viral spread [13].

Given the prevalence of PMX cases in homosexuals or men who have sex with men, the WHO stated that it is essential to practice safe sex by using condoms, which minimizes the risk of sexually transmitted diseases [64]. In addition, it is essential to be tested frequently for STIs and treated as early as possible to avoid consequences.

Governments and health policymakers must learn from prior outbreaks and take proactive measures to stop the recent worldwide spread of MPX. Furthermore, it is critical to understand the present status of MPX outbreaks worldwide, the global epidemiological and public health situation, and what can be done to prevent further spread and future global ramifications.

Finally, the prevention of PXM requires a combination of disease control measures, hygiene education and preventive measures, and a rapid and effective response in the event of an outbreak.

## 5. Conclusions

The findings of the current investigation support the theory that MPX is sexually transmitted, because MPXV was found in the seminal fluid of MPX patients. Men who have sex with other men run a higher chance of catching the illness and spreading it. The creation of hygienic standards is essential for the early identification of MPX cases.

## Data Availability

The data of the results were uploaded in Github severs as https://github.com/jbarbozameca/Tropicalmed-2098479 (accessed on 9 January 2023).

## Supplementary Materials

The following supporting information can be downloaded at: https://github.com/jbarbozameca/Tropicalmed-2098479: Table S1: Bibliographic search strategy. Table S2: Characteristics of included studies and description of case reports of monkeypox. Table S3: Cases of MPX with clinical manifestations, localization, and prognosis reported in the included studies.
